# Artificial intelligence applications in the diagnosis and treatment of bacterial infections

**DOI:** 10.3389/fmicb.2024.1449844

**Published:** 2024-08-06

**Authors:** Xiaoyu Zhang, Deng Zhang, Xifan Zhang, Xin Zhang

**Affiliations:** ^1^First Department of Infectious Diseases, The First Affiliated Hospital of China Medical University, Shenyang, China; ^2^Department of Infectious Diseases, The First Affiliated Hospital of Xiamen University, Xiamen, China

**Keywords:** bacterial infections, artificial intelligence, machine learning, diagnosis, treatment, epidemiologic surveillance

## Abstract

The diagnosis and treatment of bacterial infections in the medical and public health field in the 21st century remain significantly challenging. Artificial Intelligence (AI) has emerged as a powerful new tool in diagnosing and treating bacterial infections. AI is rapidly revolutionizing epidemiological studies of infectious diseases, providing effective early warning, prevention, and control of outbreaks. Machine learning models provide a highly flexible way to simulate and predict the complex mechanisms of pathogen-host interactions, which is crucial for a comprehensive understanding of the nature of diseases. Machine learning-based pathogen identification technology and antimicrobial drug susceptibility testing break through the limitations of traditional methods, significantly shorten the time from sample collection to the determination of result, and greatly improve the speed and accuracy of laboratory testing. In addition, AI technology application in treating bacterial infections, particularly in the research and development of drugs and vaccines, and the application of innovative therapies such as bacteriophage, provides new strategies for improving therapy and curbing bacterial resistance. Although AI has a broad application prospect in diagnosing and treating bacterial infections, significant challenges remain in data quality and quantity, model interpretability, clinical integration, and patient privacy protection. To overcome these challenges and, realize widespread application in clinical practice, interdisciplinary cooperation, technology innovation, and policy support are essential components of the joint efforts required. In summary, with continuous advancements and in-depth application of AI technology, AI will enable doctors to more effectivelyaddress the challenge of bacterial infection, promoting the development of medical practice toward precision, efficiency, and personalization; optimizing the best nursing and treatment plans for patients; and providing strong support for public health safety.

## 1 Introduction

Bacterial infections remain a major challenge in medical and public health in the 21st century, with millions of patient deaths annually. According to a study published in The Lancet on November 21, 2022, bacterial infections are one of the leading causes of global health loss and have become the second leading cause of death globally, after ischemic heart disease (GBD, 2019). Accurate and rapid identification of pathogens and their drug susceptibility profiles is essential for selecting the right treatment and reducing mortality. However, most current bacterial identification and drug susceptibility testing require culture times of several days, which not only delays the initiation of treatment, but also increases the risk of the development of resistant bacteria due to the long-term use of broad-spectrum antibiotics. At the same time, surveillance and management of bacterial infections are essential to prevent their spread and safeguard public health. In this context, the medical community urgently seeks new tools and strategies to better cope with bacterial infections. The rise of artificial intelligence (AI) technology, offers a new way to deal with bacterial infection (Mintz and Brodie, 2019; Larentzakis and Lygeros, 2021; Ting Sim et al., 2023).

**Figure 1:**
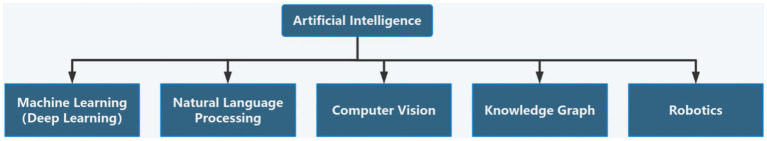
The relationship between machine learning (particularly deep learning), natural language processing, computer vision, knowledge graph, robotics, and artificial intelligence.

Recently AI, as a powerful computational tool, has shown great potential in the diagnosis and treatment of bacterial infections (Goodswen et al., 2021; Jiang et al., 2022). AI is a science and technology that simulates human intelligence through computers, capable of mimicking human cognitive abilities and decision-making processes. In medicine, the main focus should be on the following terms: machine learning (particularly deep learning), natural language processing, computer vision, knowledge graph, and robotics, etc. (Mintz and Brodie, 2019) (Figure 1). The rapid expansion of AI technology spans from enhancing epidemiological surveillance to accelerating pathogen identification and predicting bacteria sensitivity to antimicrobial agents, Furthermore, AI supports the research and development of new drugs, vaccines, and innovative therapies, thereby promoting the development advancement of personalized medicine. tThe wide application of AI is expected to fundamentally transform the management, diagnosis, and treatment of bacterial infection (Wong et al., 2023).

Based on a comprehensive analysis of the existing literature and the latest research results, this study aimed to explore how AI technology can improve the efficiency and accuracy of medical diagnosis, as well as the level of personalized treatment, while focusing on the challenges that may hinder its practical clinical application. This will primarily provide medical workers with a comprehensive understanding of the application of AI technology in the diagnosis and treatment of bacterial infectious diseases, jointly promote the application of AI in the fight against bacterial infections, provide patients with more accurate and efficient medical services, and contribute to the development of global public health.

## 2 Application of AI in epidemiological surveillance of bacterial infectious diseases

AI and big data technologies are rapidly transforming the epidemiology of infectious diseases, particularly in the research and management of public health emergencies (PHEs). The modelsof infectious disease dynamics (IDD) and dynamic Bayesian networks (DBN)have not only promoted the spread of disease forecast accuracy, but also strengthened the ability analysis outbreakevolution (Gao and Wang, 2022). Through cloud computing platforms, AI can process massive data in real time and effectively monitor infectious disease outbreaks. Despite the challenge of long model training time, its practicability makes it an indispensable tool for early epidemic warning (Li et al., 2023). In addition, the development and application of geographic information systems (GIS), with its advanced data overlay capabilities, has greatly optimized the integration of public health data and has gained widespread acceptance (Wells et al., 2021). Similarly, the ToxPi*GIS Toolkit enables the visualization and analysis of geospatial data in the ArcGIS environment, a visualization framework that integrates multiple data sources and generates intuitive graphic files with through Python scripts, ArcGIS Pro methods, and custom toolkits (Fleming et al., 2022). In addition, the cloud data storage and use of Internet search data, such as Google Flu Trends, show the potential of disease surveillance systems based on large data to enhance real-time monitoring (Pfeiffer and Stevens, 2015).

Although these advanced tools and methods are currently used primarily in viral epidemiology, their potential for disease surveillance, data presentation and analysis, and public health decision-making continues to evolve. This suggests that their contribution to bacterial epidemiology is also expected to increase. For example, machine learning models can predict in advance the risk of *Clostridioides difficile* infection among patients in large hospitals, allowing healthcare teams to implement preventive measures proactively before infection occurs (Oh et al., 2018; Tilton and Johnson, 2019). Real-time locator systems can be used for contact tracing in the emergency department, which is not only more efficient and timely than tracing methods relying on electronic medical records, but also significantly increases the number of potentially exposed individuals identified while optimizing the use of time and resources (Hellmich et al., 2017). Maia Lesosky et al. revealed the impact of inter-hospital patient flow on methicillin-resistant *Staphylococcus aureus* (MRSA) transmission through Monte Carlo simulation (Lesosky et al., 2011). Further studies explored cross-hospital pathogen transmission using a susceptible infection model, demonstrating the important value of AI and big data in curbing hospital-acquired infections (Ciccolini et al., 2014).

AI is paving new ways to predict and prevent bacterial infections. AI technology integrates and analyzes vast amounts of complex data to achieve early recognition and accurate prediction of bacterial infection outbreaks. This optimizes prevention and control measures, guides public health decisions, and supports the global fight against infectious diseases and the new solution.

## 3 AI has revolutionized the study of bacterial infection mechanism

Further study of the pathogenesis of bacterial infectious diseases is crucial to fully understand the nature of these diseases. This process not only involves the complex process of how bacteria colonize, invade, and spread in the host but also involves the host’s immune response and its interaction with pathogens. Among them, pathogen-host interaction is the key link, and animal models have been an indispensable tool in traditional research. They provide valuable data for observing the infection process of pathogens, host immune response, and disease development (Younes et al., 2020; Burkovski, 2022). While such approaches, although capable of providing accurate and rich biologic insights, are often costly, time-consuming, and associated with ethical concerns. With the rapid development of AI technology, especially the emergence of machine learning models, researchers can simulate and understand the complex interactions between pathogens and hosts without animal experiments. For example, the PHISTO tool promotes a deep understanding of infection mechanisms by synthesizing different databases and using text mining techniques, supplemented by graph theory analysis and BLAST search (Durmuş Tekir et al., 2013). A novel set of modular structural plasmids named pTBH (toolbox of Haemophilus) demonstrates coexistence and co-infection kinetics of fluorescently labeled strains by 3D microscopy combined with quantitative image analysis (Rapún-Araiz et al., 2023). Furthermore, AI models can effectively simulate the complex interactions between bacteria and hosts in different metabolic states (Dillard et al., 2023). Using advanced fluorescence microscopy detection and automated image analysis techniques, researchers have found that *Staphylococcus aureus* isolates from patients with bone/joint infection, bacteremia, and infective endocarditis show different infection characteristics in different host cell types (Rodrigues Lopes et al., 2022). These techniques not only provide a visual basis for understanding microbial behavior in specific host environments but also assist in the design of drugs and vaccines.

The application of machine learning models provides us with a highly flexible way to predict and simulate the complex mechanisms of pathogen-host interactions, which not only accelerates the research process but also reduces the research cost. Although AI models are not a complete replacement for all animal model studies, they provide new ways to explore uncharted territories.

## 4 AI application in the diagnosis of bacterial infections

**Figure 2:**
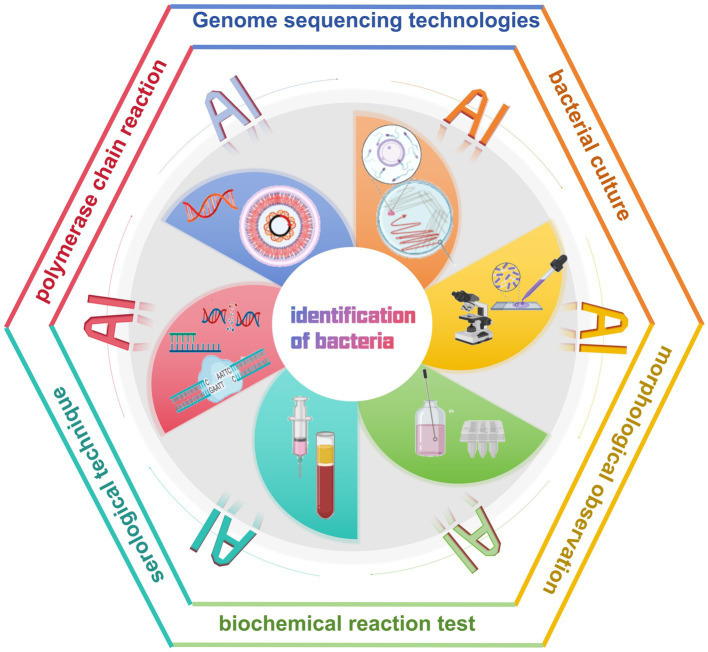
Artificial intelligence facilitates the diagnosis of bacterial infectious diseases.

In the traditional approach to diagnosing bacterial infectious diseases, laboratory technicians rely on microbiological and biochemical tests to identify pathogens. It includes bacterial culture, morphological observation, biochemical reaction tests, and serological techniques (Ernst et al., 2006; Váradi et al., 2017) (Table 1). In addition, molecular biology techniques are widely used for the identification of bacterial DNA sequences, of which the polymerase chain reaction (PCR) is a commonly used method (Wilson, 2015; Deusenbery et al., 2021). Although PCR technology is more advanced than traditional biochemical and microbiological methods, it requires a long time to complete the experimental process. Moreover, the integration and application of AI technology not only optimizes the traditional bacterial detection and management process, but also has the potential to bring about a complete revolution (Ho et al., 2019; Wang et al., 2020; Paquin et al., 2022; Howard et al., 2024) (Figure 2).

**Table 1:** Advantages and limitations of the traditional bacterial identification methods.

Method	Advantages	Limitations
Bacterial culture (Baron, 2019)	✓ The cost is low ✓ Effective for various bacteria ✓ Easy to operate	Long time consumption Some bacteria cannot develop Susceptible to contamination It is not suitable for highly specific tests
Morphological observation (Periasamy, 2014)	✓ No special equipment ✓ Intuitive is strong ✓ Accumulation of experience	Subjectivity is strong Limited information The lack of specificity Need to develop
Biochemical reaction tests (Ohkusu, 2000)	✓ Cost-effective ✓ Easy to operate	Limited specificity Does not apply to all bacteria
Serological technique (Eldin et al., 2019)	✓ High specificity ✓ Quick results ✓ Quantifiable analysis	Greatly influenced by sampling time There were false positive and false negative results A variety of pathogens have cross-reacted

### 4.1 AI improves the efficiency and accuracy of pathogen identification

AI technology provides a new way to diagnose bacterial infections rapidly and accurately. For example, matrix-assisted laser desorption/ionization time-of-flight mass spectrometry (MALDI-TOF MS) combined with ClinProTools software provided a method for the rapid identification of two *Staphylococcus aureus* subspecies, which achieved 100% identification and classification accuracy through genetic analysis and a fast classifier model (Pérez-Sancho et al., 2018). Findaureus, an open-source application based on Python, demonstrated the ability to automatically locate bacteria in the tissue section using immune fluorescent tags. It overcomes the challenges of the manual threshold-setting process and optimizes the analysis of the condition of complex tissue cell efficiency (Mandal et al., 2024). PhenoMatrix (PM) Colorimetric Detection Module (CDM) digital imaging software uses the automated Walk Away Specimen Processor to detect Group B Streptococcus (with high sensitivity similar to that of molecular testing methods, increasing laboratory productivity and reducing the potential for human error (Baker et al., 2020). In addition, DNA microarray technology, using a machine learning decision-making algorithm (DendrisChips), identifies 11 types of bacteria associated with respiratory tract infections within 4 h. This technology combines PCR amplification of bacterial 16S rDNA and specific oligonucleotide hybridization on DendrisChips®, which are read with a laser scanner, thereby achieving quick and accurate detection and differentiation with over 95% accuracy (Senescau et al., 2018). Using neural networks to analyze response patterns, a researcher has designed a sensor capable of identifying 16 different bacterial species and their Gram-staining properties with >90% accuracy. The sensor is stable for up to 6 months after preparation and requires one-thirtieth the amount of dye and sample as traditional solution-based sensors, compared to conventional techniques (Laliwala et al., 2022). Thus, this method provides an innovative diagnostic tool that promises clinical applications in resource-limited settings.

In diagnosing diseases that pose a serious threat to human health, such as tuberculosis, although conventional microscopy methods are effective, they are slow and of limited sensitivity. The introduction of AI, specifically Metasystems’ automated antifungal bacilli (AFB) smear microscopy scanning and deep learning-based image analysis module (Neon Metafer), has greatly improved the speed and accuracy of antifungal bacilli (AFB) smear-negative slide recognition speed and accuracy (Desruisseaux et al., 2024). A deep neural network (DNN) classifier combined with an automated slide scanning system reduces analysis time from several minutes to approximately 10 s per slide (Horvath et al., 2020). Further, a novel diagnostic system combining T-SPOT with DL-based computed tomography image analysis can significantly improve the classification accuracy of nontuberculous mycobacterial lung disease and pulmonary tuberculosis (Ying et al., 2022). AI tools, such as artificial neural networks, are becoming important in providing rapid and effective pathogen detection methods (Dande and Samant, 2018). AI technology brings unprecedented accuracy and speed to pathogen detection through efficient learning and analysis capabilities. It will not only promote the automation of pathogen detection but also substantially decrease error rates caused by human operation, thereby improving the reliability of the diagnostic process.

### 4.2 AI optimizes antimicrobial susceptibility testing

Identifying pathogens and performing Antimicrobial Susceptibility Testing (AST) in today’s clinical laboratories often relies on culturing and isolating pathogens. Standard AST methods (CLSI, 2023 such as disk diffusion, microbroth dilution, and AGAR dilution methods, typically require 2–3 days or longer from sample collection to obtaining culture and drug susceptibility results (Abu-Aqil et al., 2023). To effectively control infections and prevent them from rapidly deteriorating or spreading to other parts of the body, clinicians often choose broad-spectrum antimicrobials for empirical treatment, given that many infectious diseases are often difficult to diagnose by symptoms in the early stages. However, this practice may increase the risk of drug-resistant strains arising due to inappropriate drug selection; therefore, there is an urgent need for rapid and accurate AST technologies to guide diagnosis and treatment.

With the rapid advancement of technology, AI has become an important tool in bacterial AST, providing various efficient and rapid methods to perform drug susceptibility testing. For example, Raman spectroscopy based on image stitching technology enables single-cell level detection, which can automatically, efficiently, and rapidly identify drug-resistant bacteria (Nakar et al., 2022; Dou et al., 2023). Combining machine learning and infrared spectroscopy enables rapid and definitive identification of urinary tract infection bacteria and their drug resistance, dramatically reducing the time from sample collection to results. This approach decreases the time of identification and sensitization of *Escherichia coli*, *Proteus mirabilis*, and *Pseudomonas aeruginosa* from 48 h to approximately 40 min (Ciccolini et al., 2014; Tilton and Johnson, 2019; Younes et al., 2020; Burkovski, 2022). Similarly, the SlipChip microfluidic device uses electrophoresis technology to extract and enrich bacteria directly from positive blood cultures. This device enables parallel inoculation of bacteria into nanoscale droplets of broth, facilitating simultaneous multiple AST. Results can be reported to clinicians within 3–8 h, ensuring reliable AST results and enabling earlier reporting and targeted antimicrobial treatment (Yi et al., 2019).

Automation technology has also demonstrated high efficiency in detecting certain special drug-resistant bacteria, such as MALDI-TOF MS, for the detection of MRSA and carbapenem-resistant *Klebsiella pneumoniae* (CRKP) (Wieser et al., 2012; Zhang et al., 2023). However, the novel ML-based MALDI-TOF MS method enables rapid identification of MRSA and CRKP from labeled blood cultures within 1 h (Yu et al., 2023a,b). Recent studies have shown that using computer science to analyze a large number of MALDI-TOF MS data can provide a comprehensive understanding of western blot mapping between resistant and sensitive isolates (Wang et al., 2021). WASPLab automation system can significantly shorten the vancomycin resistant enterococcus (VRE) recognition time (Cherkaoui et al., 2019). In addition, the automated plate evaluation system (APAS Independence) has significantly improved the productivity of high-throughput laboratories through its highly sensitive digital image analysis technology to accurately classify MRSA and sensitive *Staphylococcus aureus* (MSSA) cultures as negative or positive without human intervention (Gammel et al., 2021).

In conclusion, the application of AI technologies to antimicrobial susceptibility testing enables the rapid and accurate identification of drug-resistant bacteria, thereby dramatically shortening the time from sample collection to result confirmation, and can be accomplished without human intervention. These technologies provide laboratories with a rapid and automated means of drug resistance monitoring, which significantly improves diagnostic efficiency and helps clinicians make rational antimicrobial treatment decisions as early as possible (Table 2).

**Table 2:** Artificial intelligence in the bacteria identification and drug sensitivity analysis.

Technology	Application	References
MALDI-TOF MS + ClinProTools software	Rapidly identified *Staphylococcus aureus* subspecies	Pérez-Sancho et al. (2018)
Findaureus	Automatic localization of bacteria in immunofluorescently labeled tissue sections	Mandal et al. (2024)
PM + CDM + WASP	High sensitivity to identify group B streptococcus	Baker et al. (2020)
Machine learning-based DNA micro-matrix technology	More than 95% accuracy in identifying respiratory bacteria	Senescau et al. (2018)
Neural network-based sensors	90% accuracy in bacterial identification	Laliwala et al. (2022)
AFB + Neon Metafer	Significantly improved the speed and accuracy of identification of acid-fighting bacilli (AFB) on smear-negative slides	Desruisseaux et al. (2024)
DNN + an automated slide scanning system	Significantly reduced slide analysis time	Horvath et al. (2020)
T-SPOT + DL-based technology	Significantly improved the classification accuracy of NTM—PD and PTB	Ying et al. (2022)
Raman spectroscopy based on image stitching technology	Automatically, efficiently and rapidly identified drug-resistant bacteria	Dou et al. (2023) and Nakar et al. (2022)
SlipChip microfluidic device	Significant reduction in bacterial drug sensitivity test time	Yi et al. (2019)
A novel MALDI-TOF MS method based on ML	Rapidly identified MRSA and CRKP	Yu et al. (2023a,b)
WASPLab automation system	Significantly shorten the vancomycin-resistant enterococcus (VRE) recognition time	Cherkaoui et al. (2019)
APAS Independence	Accurately distinguish MRSA and MSSA	Gammel et al. (2021)

### 4.3 AI can improve bacterial genome sequencing

Genome sequencing technologies (including whole genome sequencing and next-generation sequencing) have significantly accelerated not only the identification of infectious agents, but also the tracking of transmission pathways in healthcare settings and the analysis of the impact of complex microbial communities on human health (d’Humières et al., 2021; Deusenbery et al., 2021). It also provides a powerful tool for monitoring and responding to antimicrobial resistance (AMR) globally (Waddington et al., 2022; Sherry et al., 2023).

Current genetic testing techniques mainly match based on sequence similarity; however, these tools are often unsuccessful in identifying new species without closely related genomes or related sequences in reference databases. In response to this challenge, the machine learning-based PaPrBaG method provides a reliable and consistent prediction method that maintains its reliability even with low genome coverage (Deneke et al., 2017). In addition, machine learning combined with metagenomic sequencing can significantly improve the diagnostic accuracy of diseases that are difficult to diagnose, such as tuberculous meningitis (Ramachandran et al., 2022).

Another challenge for genetic testing technologies is how to rapidly and accurately interpret high-dimensional genomic data as the cost of second-generation sequencing technology decreases and throughput increases. Machine learning techniques have shown their potential in processing large genomic data by analyzing and predicting the health impact of Shiga toxin-producing *Escherichia coli* infections, providing new methods and perspectives for microbial risk assessment (Njage et al., 2019). In addition, Bayesian neural networks using a nonparametric Bayesian algorithm excelled in accelerating the analysis of genetic association studies and efficiently and accurately identifying variant strains of infection (Beam et al., 2014).

Combining machine-learning models with genomics technology has shown excellent performance in predicting pathogen resistance, which is significantly better than existing methods. Some researchers have used machine learning to construct a knowledge map of antimicrobial resistance in *Escherichia coli*, which realizes the automatic discovery of knowledge of antimicrobial resistance in *Escherichia coli* and reveals unknown drug resistance genes (Youn et al., 2022). Based on the XGBoost and convolutional neural network approaches, the researchers not only accurately predicted the minimum inhibitory concentrations of *Klebsiella pneumoniae* clinical isolates against 20 antimicrobial drugs, but also successfully identified strains with high drug resistance or high virulence (Nguyen et al., 2018; Liu et al., 2021; Lu et al., 2022). Similarly, some researchers have innovated a decision tree method called Treesist-TB for identifying mutant strains and predicting drug resistance, which has a recognition ability beyond the existing TB-Profiler tools (Deelder et al., 2022), This technique not only demonstrates the value of decision trees in the tuberculosis field but also provides a reference template to identify other drug-resistant pathogens.

AI has shown great potential in genome sequencing technology. In response to the challenges of identifying new species and interpreting high-dimensional data, machine learning has surpassed the limitations of traditional genetic detection techniques and deepened our understanding of the microscopic world of pathogens. Furthermore, machine learning excels in predicting antimicrobial drug resistance, outperforming traditional methods, and strengthening global antibiotic resistance (AMR) monitoring efforts.

## 5 Application of AI in the treatment of bacterial infections

The challenges in the treatment of bacterial infections are diverse, and one of the most serious is the increasing resistance to antimicrobial agents. The importance of Antimicrobialresistance was formally declared at the United Nations General Assembly High-level Meeting on antimicrobial Resistance in 2016, and countries were called on to commit to developing their national action plans on antimicrobial resistance. Nearly 5 million people died globally due to resistant pathogens in 2019 (Antimicrobial Resistance Collaborators, 2022). Current projections suggest that by 2050, 10 million people globally could be burdened by antimicrobial drug resistance each year (Walsh et al., 2023). Over time, bacteria have acquired resistance to antimicrobial drugs through natural selection and genetic variation, thereby undermining the effectiveness of traditional treatments. In addition, the high diversity of bacteria and the complexity of bacterial-host interactions further increase the difficulty of treatment, making the development of vaccines and novel drugs difficult. Hence, developing new antimicrobial strategies and therapeutic approaches are urgently needed to address these issues (Stracy et al., 2022).

**Figure 3:**
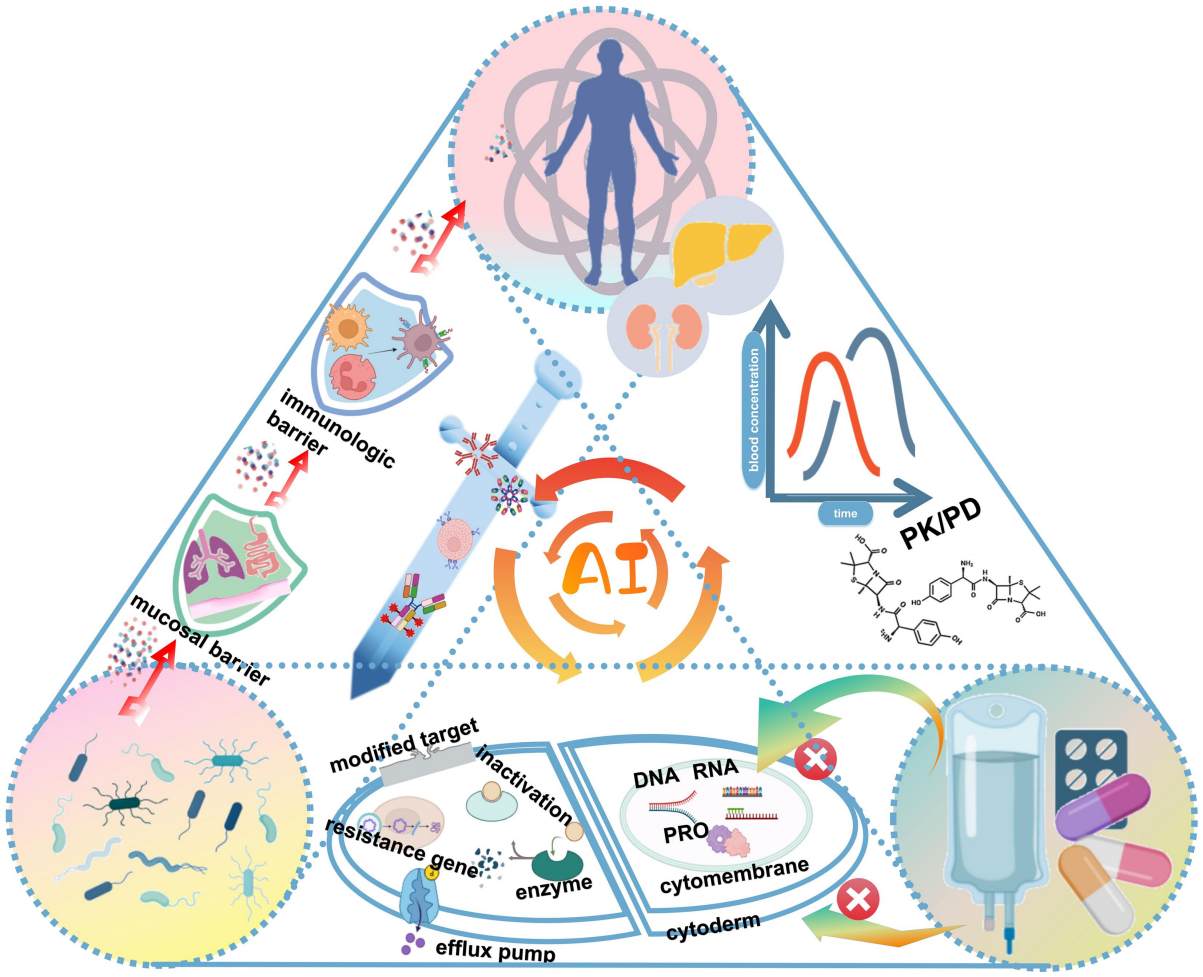
AI technology can model complex interactions between pathogens, hosts, and drugs.

In this context, AI technology accurately simulates the complex interactions between pathogen, host, and drugs, revealing microbial infection features and optimizing drug and vaccine design (Figure 3). In addition, AI application in the field of phage therapy brings new hope for the fight against bacterial resistance.

### 5.1 AI revolutionizes drug discovery and development

In drug research and development, the application of AI is breaking the boundaries of traditional research, providing new strategies to overcome the problem of drug resistance. For example, by combining high-throughput biophysical analysis and machine learning, a framework was established to identify and predict bioactive targets of antimicrobial drugs, which successfully revealed the relationship between phenotype, target, and chemotype, providing an effective way to identify candidate therapeutic drugs (Santa Maria et al., 2017). Meanwhile, combining fragment-based drug design with quantitative structure–activity relationship modeling demonstrates the potential of artificial neural networks in the drug discovery process (Kleandrova and Speck-Planche, 2020). Using data-driven techniques, the study of bacterial minimal inhibitory concentration data using machine learning and matched molecular pair analysis has revealed key chemical features that affect bacterial biological activity, thus promising to expand the chemical space of broad-spectrum antimicrobial agents (Gurvic et al., 2022). In a study, a support vector machine learning approach was applied to analyze genomics, metabolomics, and transcriptomics data of *Pseudomonas aeruginosa*. This approach successfully identified a key molecular mechanism that distinguishes between pathogenic and non-pathogenic strains of *Pseudomonas aeruginosa*, which not only provides high-value targets for the development of novel antimicrobial therapeutics but also highlights the importance of dynamically integrating multidimensional data in modern drug discovery and development (Larsen et al., 2014).

Furthermore, significant breakthroughs have been made in the application of AI in specific disease areas, such as anti-tuberculosis drug development. The machine learning and artificial neural network method can be used to successfully find LeuRS for *Mycobacterium tuberculosis* and MetRS double targets of inhibitors (Volynets et al., 2022), and small-molecule inhibitors of the enzymes required for *M. tuberculosis* topoisomerase I have been successfully identified (Ekins et al., 2017), providing a new strategy to overcome multidrug resistance in tuberculosis. In addition, combining public Mtb data with machine learning not only greatly improves the efficiency of drug discovery, but also accumulates valuable data resources for future anti-tuberculosis research and new drug development (Lane et al., 2022).

These advanced technologies not only accelerate the research and development process of new drugs, but also enhance the possibility of discovering potential therapeutic options, fundamentally changing how researchers understand and operate complex biological systems, and heralding a new era of smarter and more precise development in the pharmaceutical field.

### 5.2 AI brings breakthroughs in vaccine development

Currently rapid progress has been made in vaccine research and development against viral diseases. In particular, the speed and efficiency of response to emerging virus epidemics have been greatly improved, such as the application of computer-aided design of COVID-19 vaccine candidates in the global pandemic of COVID-19 in early 2020 (Abbasi et al., 2022). In contrast, bacteria in the field of vaccine research and development are faced with more complicated challenges. The high variability of bacteria, rapidly evolving drug resistance, and complexity of interactions between bacteria and their hosts all challenge the development of effective vaccines against bacterial infectious diseases. To address these challenges, leveraging emerging tools such as artificial intelligence, computer-aided design, and advanced immunological evaluation techniques has become pivotal to accelerating the development of safe and effective vaccines.

In the process of vaccine design, scientists are challenged not only to identify the key antigens that can trigger lasting immune memory, but also to ensure that the vaccine can elicit broad protective immune responses, including humoral and cellular immune responses, to achieve effective protection in the long term. Recently, reverse vaccinology (RV) technology has been widely used in vaccine research and development. As a calculation method, RV is mainly applied to bacterial pathogens. Bexsero, a *Neisseria meningitidis* B vaccine designed by RV, has been registered and widely used in many countries (Heinson et al., 2015). In addition, a key component of vaccine development—antigen identification—is strongly supported by computational tools such as deep learning, reverse vaccinology and immunoinformatics. In-depth analysis of vaccine targets derived from pathogen protein-coding genomes has led to the successful development of a multi-epitope subunit vaccine with potentially potent protection. Although the safety and immunogenicity of the vaccine need to be further verified (Rawal et al., 2021), this approach not only accelerates the vaccine design process and reduces the reliance on traditional trial methods, but also has important implications for addressing the threat of drug-resistant bacteria. Research shows that a new type of machine learning model, compared with traditional methods, achieves higher precision and sensitivity in predicting aspects of *mycobacterium tuberculosis* (Khanna and Rana, 2019).

The application of machine learning technology not only optimizes the vaccine development process and improves efficiency by reducing the reliance on traditional experiments and animal testing, but also provides strong scientific and technological support to cope with evolving epidemics of bacterial infections.

### 5.3 AI drives innovative applications of phage therapy

Phage therapy has attracted much attention from the scientific community for its potential advantages in combating drug-resistant bacterial infections (Viertel et al., 2014; Kulshrestha et al., 2024). However, accurate prediction of the complex interactions between phages and their target pathogens and hosts remains challenging (Cisek et al., 2017), and AI models become an important tool to overcome this challenge. For example, a machine learning-based local K-mer strategy is used to accurately predict phage-bacteria interactions (Qiu et al., 2024). Simultaneously, machine learning can assist in the design of clinical phage therapy, particularly for urinary tract infections caused by multidrug-resistant *E. coli* (Keith et al., 2024). In addition, a tool called HostPhinder predicted phage host genus and species with 81 and 74% accuracy, respectively, demonstrating the technology’s ability to pinpoint therapeutic targets (Villarroel et al., 2016).

Consequently, the application of phages, either alone or in combination with antimicrobial agents, can be a viable alternative to treat infections with resistant pathogens (Tagliaferri et al., 2019). The rapid development of AI technology enhances the potential of phage therapy by accurately predicting complex interactions between pathogens and phages, thereby contributing to the design of personalized treatment. This not only accelerates the development of phage therapy but also enhances its treatment success.

### 5.4 AI-assisted clinical decision support systems

The timing of effective antimicrobials is a key determinant of morbidity and mortality in the management of infectious diseases, specifically in the case of septic shock (Evans et al., 2021). Early identification can not only reduce the poor prognosis caused by delayed treatment, but also help avoid unnecessary medical intervention and reduce treatment costs, thus significantly improving the survival rate and quality of life of patients.

Under the background of increasing emphasis on individualized treatment and precision medicine, AI progress not only promotes medical innovation, but also may overturn the existing diagnosis and treatment mode. In bacterial infectious disease diagnosis and treatment, AI and ML are used to simplify the clinicians’ work process, improve the quality of decision-making, and promote the development of personalized treatment options (Langford et al., 2024). For example, ML models have been successfully applied to diagnose respiratory syncytial virus infection and pertussis in children by combining clinical symptoms with laboratory test results (Mc Cord-De Iaco et al., 2023). Based on statistically significant clinical indicators such as sex and age, LightGBM and other ML models have a good effect on predicting the etiology of classical Fever of Unknown Origin in patients (Yan et al., 2021). In addition, ML models can rapidly predict the risk of MRSA infection in patients with community-acquired pneumonia and facilitate the implementation of targeted antimicrobial treatment (Rhodes et al., 2023). Clinical decision trees generated based on recursive methods are valuable for determining the likelihood of infection with extended-spectrum beta-lactamase strains in patients with bacteremia (Goodman et al., 2016). A system for early warning of antimicrobial drug allergies, K-CDSTM, effectively warns of antimicrobial drug allergies and prevents patients from being prescribed antimicrobial drugs that may trigger allergic reactions (Han et al., 2024). The ontology-driven clinical decision support system uses big data to assist the treatment decision-making of infectious diseases and constructs a bridge between patients and medical workers (Shen et al., 2018). In the development of predictive disease models, tools such as multiple infectious disease diagnostic models are significantly more accurate than traditional prediction techniques based on large amounts of training data (Wang et al., 2022). In a 3-month case–control study using a computerized clinical decision support system in an experimental group, time was reduced by approximately 1 h and antimicrobial costs were saved by approximately US $84,000 (McGregor et al., 2006).

**Figure 4:**
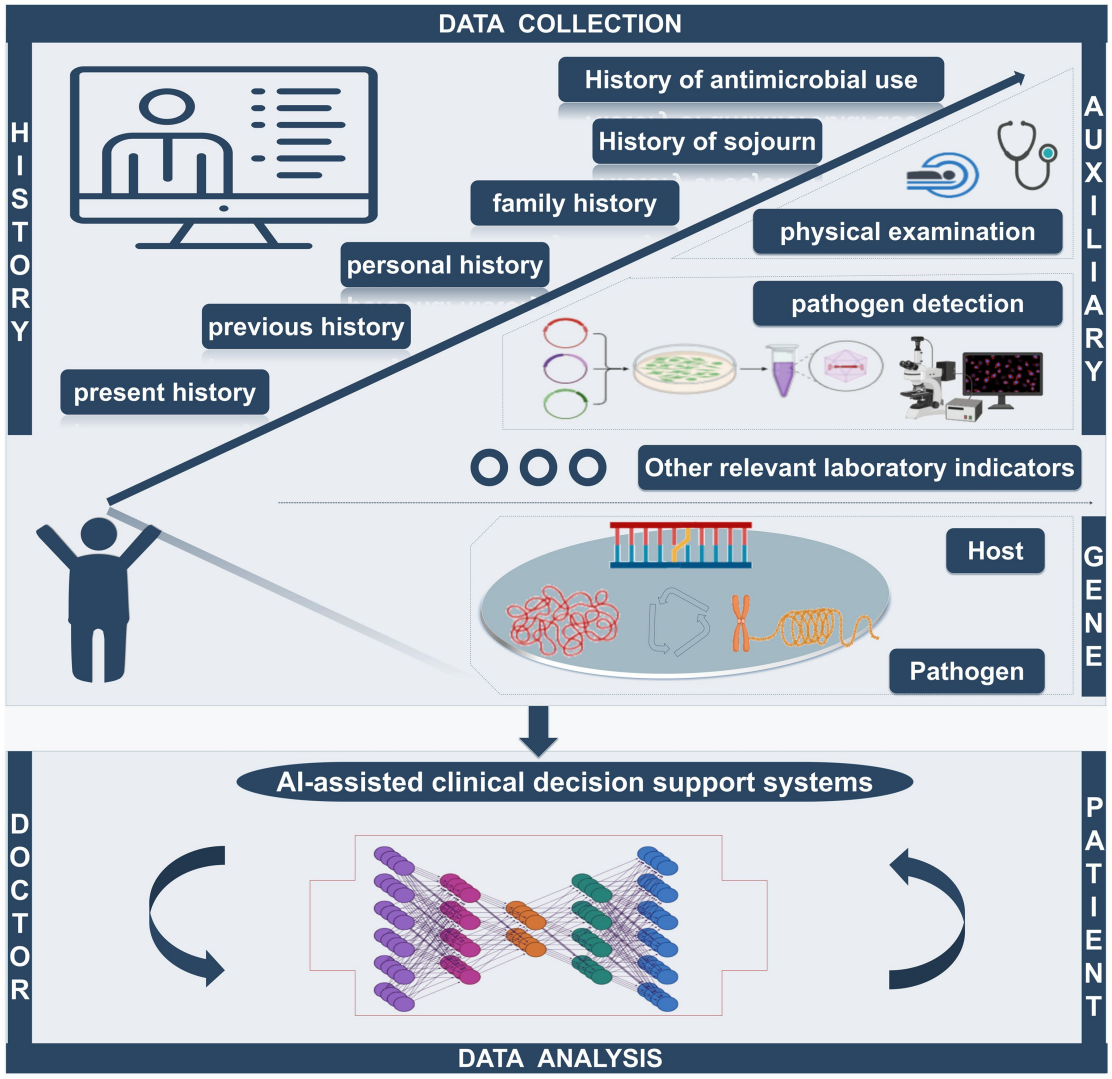
The AI-assisted clinical decision support system can quickly collect the patient’s history of present disease, past history, personal history, family history, travel history, and antibiotic use history. Simultaneously, the system can integrate relevant auxiliary examination (including imaging examination and laboratory examination) and analysis of the genetic information of hosts and pathogens to provide the best treatment, becoming a bridge of effective communication between doctors and patients.

In summary, machine learning models have been successfully used to improve diagnostic accuracy and predict disease risk in clinical decision-making, showing better accuracy and efficiency than traditional approaches (Figure 4). AI and ML technologies are leading the wave of medical innovation and have the potential to change the traditional methods of diagnosis and treatment.

## 6 AI helps personalized medical development

Through deep study and the analysis of the complex algorithm, AI can process and interpret patients with huge amounts of data, including genetic information, living habits and historical health records, etc. This not only enables accurate diagnosis of the disease, but also facilitates personalized treatment plans for each patient. For example, in cancer treatment, AI can help doctors choose the most appropriate combination of drugs for patients, reduce side effects, and improve cure rates. Similarly, AI can also predict efficacy and possible complications and provide tailored health management plans for patients (Bilgin et al., 2024; Elemento, 2024).

In the field of bacterial infections, a novel method called CombiANT can rapidly quantify antimicrobial synergy through a single test and automated image analysis, enabling personalized clinical synergy testing to improve the anti-infection combination therapy (Fatsis-Kavalopoulos et al., 2020). Kuo-Wei Hsu et al. developed an automated portable antimicrobial susceptibility testing system for four common urinary tract infection bacterial strains, taking only 4.5–9 h to complete the test, which holds promise for future application in personalized medicine practice (Hsu et al., 2021). Connor Rees et al. showed an overall success rate of > 90% for correct diagnoses in the list of 10 differential diagnoses generated by ChatGP-3 (Hirosawa et al., 2023). In the future, more research is expected to focus on evaluating more complex cases and promote the development of fully trained artificial intelligence chatbots to improve the accuracy and completeness of diagnosis and further personalize patient treatment.

## 7 Challenges of AI in the medical field

**Figure 5:**
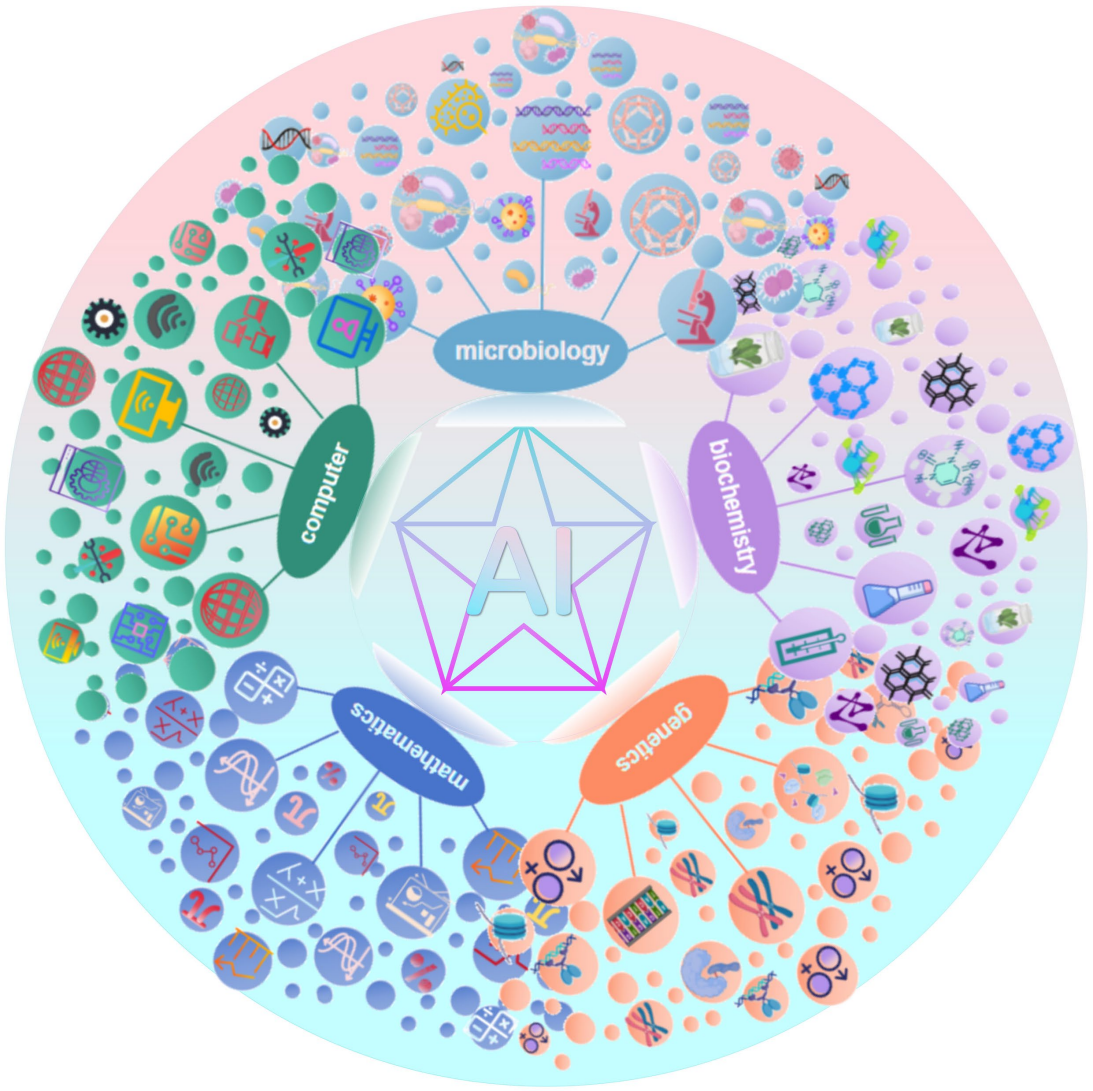
The successful application of AI models in medicine relies on multidisciplinary collaboration.

Although the application of AI in the field of bacterial infections has great potential and prospects, it also faces numerous challenges. The first is the problem of data quantity and data quality. The collection, sorting and sharing of case data related to bacterial infectious diseases are restricted by privacy protection and standardization, which limits the training efficiency and application scope of AI models (Cath, 2018; Baowaly et al., 2019; Hummel and Braun, 2020). Second, deep-learning algorithms often lack the ability to provide a convincing explanation for their predictions—the so-called “black box” problem—which can affect prediction accuracy and public trust in AI systems (Schwartz et al., 2024). In addition, most healthcare AI research to date has been done in non-clinical Settings, with few instances of successful integration of AI into clinical care and most cases are still in the experimental stage (Alami et al., 2020). Therefore, generalizing the results of the study may be challenging. Moreover, complex and variable bacterial infection mechanisms and rapid mutation of bacterial genes make it more difficult to accurately predict pathogen behavior and drug sensitivity. Furthermore, the establishment of AI models requires interdisciplinary fields, including microbiology, biochemistry, genetics, mathematics and computer science, etc. (Figure 5), This requires a high level of knowledge and skills from the researchers and developers, posing a significant challenge for research teams with limited resources.

Currently in the field of artificial intelligence, a perfect legal system and authoritative standards have not been established. With the continuous progress of technology and the expansion of application fields, formulating and updating relevant regulations is essential, which will be a dynamic development process (Rees and Müller, 2022).

## 8 Conclusion

The rise of AI technology has opened up a new way to deal with bacterial infections. With the help of advanced technologies such as machine learning and deep learning, AI has been applied in many key areas, from rapid pathogen detection and antimicrobial susceptibility analysis to the interpretation of complex genomic data and the development of personalized treatment options. Through highly optimized algorithms, AI technology not only greatly improves the speed and accuracy of pathogen identification, but also accurately predicts the susceptibility of pathogens to specific antibiotics based on historical data, thus providing strong scientific decision support for doctors. Similarly, in the field of epidemiological surveillance, AI technology has strengthened the real-time monitoring and early warning ability of the spread of bacterial infectious diseases by analyzing and processing a large amount of epidemiological dataand providing a powerful analytical tool and basis for public health decision-making.

Although AI has a broad application prospect in the treatment of bacterial infectious diseases, there remain important issues to be solved, such as how to ensure the transparency and interpretability of AI decision-making and how to accelerate the diagnosis and treatment while strictly controlling the ethics and patient safety. To overcome these challenges and achieve its wide application in clinical practice, interdisciplinary cooperation, technological innovation and policy support are needed.

Prospectively, AI technology will bring a profound transformation in the field of diagnosis and treatment of bacterial infections. With the continuous strengthening and maturity of AI in pathogen identification, drug susceptibility testing and genomic analysis, it will become the right hand of clinicians. With the assistance of AI, medical workers will can better cope with the challenges brought by bacterial infections, continue to promote the development of medical practice in the direction of more precision, efficiency, and personalization, and ultimately achieve the goal of providing optimal care and treatment for patients.

## Author contributions

XYZ: Conceptualization, Data curation, Formal analysis, Investigation, Methodology, Project administration, Software, Supervision, Validation, Visualization, Writing – original draft, Writing – review & editing. DZ: Data curation, Formal analysis, Software, Supervision, Validation, Visualization, Writing – review & editing. XFZ: Data curation, Investigation, Software, Supervision, Visualization, Writing – review & editing. XZ: Conceptualization, Formal analysis, Project administration, Resources, Software, Supervision, Visualization, Writing – original draft, Writing – review & editing.

## References

[ref1] Abbasi B. A. Saraf D. Sharma T. Sinha R. Singh S. Sood S.. (2022). Identification of vaccine targets & design of vaccine against SARS-CoV-2 coronavirus using computational and deep learning-based approaches. PeerJ 10:e13380. doi: 10.7717/peerj.13380, PMID: 35611169 PMC9124463

[ref2] Abu-Aqil G. Lapidot I. Salman A. Huleihel M. (2023). Quick detection of Proteus and Pseudomonas in patients’ urine and assessing their antibiotic susceptibility using infrared spectroscopy and machine learning. Sensors (Basel) 23:8132. doi: 10.3390/s23198132, PMID: 37836961 PMC10575053

[ref3] Alami H. Lehoux P. Denis J.-L. Motulsky A. Petitgand C. Savoldelli M.. (2020). Organizational readiness for artificial intelligence in health care: insights for decision-making and practice. J. Health Organ. Manag. 35, 106–114. doi: 10.1108/JHOM-03-2020-0074 33258359

[ref4] Antimicrobial Resistance Collaborators (2022). Global burden of bacterial antimicrobial resistance in 2019: A systematic analysis. Lancet 399, 629–655. doi: 10.1016/S0140-6736(21)02724-0, PMID: 35065702 PMC8841637

[ref5] Baker J. Timm K. Faron M. Ledeboer N. Culbreath K. (2020). Digital image analysis for the detection of group B Streptococcus from ChromID Strepto B medium using PhenoMatrix algorithms. J. Clin. Microbiol. 59, e01902–e01919. doi: 10.1128/JCM.01902-19, PMID: 33087433 PMC7771474

[ref6] Baowaly M. K. Lin C.-C. Liu C.-L. Chen K.-T. (2019). Synthesizing electronic health records using improved generative adversarial networks. J. Am. Med. Inform. Assoc. 26, 228–241. doi: 10.1093/jamia/ocy142, PMID: 30535151 PMC7647178

[ref7] Baron E. J. (2019). Clinical microbiology in Underresourced settings. Clin. Lab. Med. 39, 359–369. doi: 10.1016/j.cll.2019.05.001, PMID: 31383262

[ref8] Beam A. L. Motsinger-Reif A. Doyle J. (2014). Bayesian neural networks for detecting epistasis in genetic association studies. BMC Bioinformatics 15:368. doi: 10.1186/s12859-014-0368-0, PMID: 25413600 PMC4256933

[ref9] Bilgin G. B. Bilgin C. Burkett B. J. Orme J. J. Childs D. S. Thorpe M. P.. (2024). Theranostics and artificial intelligence: new frontiers in personalized medicine. Theranostics 14, 2367–2378. doi: 10.7150/thno.94788, PMID: 38646652 PMC11024845

[ref10] Burkovski A. (2022). Host–pathogen interaction 3.0. Int. J. Mol. Sci. 23:12811. doi: 10.3390/ijms232112811 36361600 PMC9659195

[ref11] Cath C. (2018). Governing artificial intelligence: ethical, legal and technical opportunities and challenges. Philos. Trans. A Math. Phys. Eng. Sci. 376:20180080. doi: 10.1098/rsta.2018.0080 30322996 PMC6191666

[ref12] Cherkaoui A. Renzi G. Charretier Y. Blanc D. S. Vuilleumier N. Schrenzel J. (2019). Automated incubation and digital image analysis of chromogenic media using Copan WASPLab enables rapid detection of vancomycin-resistant Enterococcus. Front. Cell. Infect. Microbiol. 9:379. doi: 10.3389/fcimb.2019.00379, PMID: 31781516 PMC6851235

[ref13] Ciccolini M. Donker T. Grundmann H. Bonten M. J. M. Woolhouse M. E. J. (2014). Efficient surveillance for healthcare-associated infections spreading between hospitals. Proc. Natl. Acad. Sci. USA 111, 2271–2276. doi: 10.1073/pnas.1308062111, PMID: 24469791 PMC3926017

[ref14] Cisek A. A. Dąbrowska I. Gregorczyk K. P. Wyżewski Z. (2017). Phage therapy in bacterial infections treatment: one hundred years after the discovery of bacteriophages. Curr. Microbiol. 74, 277–283. doi: 10.1007/s00284-016-1166-x, PMID: 27896482 PMC5243869

[ref15] d’Humières C. Salmona M. Dellière S. Leo S. Rodriguez C. Angebault C.. (2021). The potential role of clinical metagenomics in infectious diseases: therapeutic perspectives. Drugs 81, 1453–1466. doi: 10.1007/s40265-021-01572-4, PMID: 34328626 PMC8323086

[ref16] Dande P. Samant P. (2018). Acquaintance to artificial neural networks and use of artificial intelligence as a diagnostic tool for tuberculosis: a review. Tuberculosis (Edinb.) 108, 1–9. doi: 10.1016/j.tube.2017.09.006, PMID: 29523307

[ref17] Deelder W. Napier G. Campino S. Palla L. Phelan J. Clark T. G. (2022). A modified decision tree approach to improve the prediction and mutation discovery for drug resistance in *Mycobacterium tuberculosis*. BMC Genomics 23:46. doi: 10.1186/s12864-022-08291-4, PMID: 35016609 PMC8753810

[ref18] Deneke C. Rentzsch R. Renard B. Y. (2017). PaPrBaG: a machine learning approach for the detection of novel pathogens from NGS data. Sci. Rep. 7:39194. doi: 10.1038/srep39194, PMID: 28051068 PMC5209729

[ref19] Desruisseaux C. Broderick C. Lavergne V. Sy K. Garcia D.-J. Barot G.. (2024). Retrospective validation of MetaSystems’ deep-learning-based digital microscopy platform with assistance compared to manual fluorescence microscopy for detection of mycobacteria. J. Clin. Microbiol. 62:e0106923. doi: 10.1128/jcm.01069-23, PMID: 38299829 PMC10935628

[ref20] Deusenbery C. Wang Y. Shukla A. (2021). Recent innovations in bacterial infection detection and treatment. ACS Infect. Dis. 7, 695–720. doi: 10.1021/acsinfecdis.0c00890 33733747

[ref21] Dillard L. R. Glass E. M. Lewis A. L. Thomas-White K. Papin J. A. (2023). Metabolic network models of the Gardnerella Pangenome identify key interactions with the vaginal environment. mSystems 8:e0068922. doi: 10.1128/msystems.00689-22, PMID: 36511689 PMC9948698

[ref22] Dou X. Yang F. Wang N. Xue Y. Hu H. Li B. (2023). Rapid detection and analysis of Raman spectra of Bacteria in multiple fields of view based on image stitching technique. FBL 28:249. doi: 10.31083/j.fbl2810249, PMID: 37919069

[ref23] Durmuş Tekir S. Çakır T. Ardıç E. Sayılırbaş A. S. Konuk G. Konuk M.. (2013). PHISTO: pathogen–host interaction search tool. Bioinformatics 29, 1357–1358. doi: 10.1093/bioinformatics/btt137, PMID: 23515528

[ref24] Ekins S. Godbole A. A. Kéri G. Orfi L. Pato J. Bhat R. S.. (2017). Machine learning and docking models for *Mycobacterium tuberculosis* topoisomerase I. Tuberculosis (Edinb.) 103, 52–60. doi: 10.1016/j.tube.2017.01.005 28237034

[ref25] Eldin C. Parola P. Raoult D. (2019). Limitations of diagnostic tests for bacterial infections. Med. Mal. Infect. 49, 98–101. doi: 10.1016/j.medmal.2018.12.004 30686500

[ref26] Elemento O. (2024). How artificial intelligence unravels the complex web of Cancer drug response. Cancer Res. 84, 1745–1746. doi: 10.1158/0008-5472.CAN-24-1123, PMID: 38588311

[ref27] Ernst D. Bolton G. Recktenwald D. Cameron M. J. Danesh A. Persad D.. (2006). “Bead-based flow Cytometric assays: a multiplex assay platform with applications in diagnostic microbiology” in Advanced techniques in diagnostic microbiology (Boston, MA: Springer US), 427–443.

[ref28] Evans L. Rhodes A. Alhazzani W. Antonelli M. Coopersmith C. M. French C.. (2021). Surviving sepsis campaign: international guidelines for management of sepsis and septic shock 2021. Intensive Care Med. 47, 1181–1247. doi: 10.1007/s00134-021-06506-y, PMID: 34599691 PMC8486643

[ref29] Fatsis-Kavalopoulos N. Roemhild R. Tang P.-C. Kreuger J. Andersson D. I. (2020). CombiANT: antibiotic interaction testing made easy. PLoS Biol. 18:e3000856. doi: 10.1371/journal.pbio.3000856, PMID: 32941420 PMC7524002

[ref30] Fleming J. Marvel S. W. Supak S. Motsinger-Reif A. A. Reif D. M. (2022). ToxPi*GIS toolkit: creating, viewing, and sharing integrative visualizations for geospatial data using ArcGIS. J. Expo. Sci. Environ. Epidemiol. 32, 900–907. doi: 10.1038/s41370-022-00433-w, PMID: 35474345 PMC9039976

[ref31] Gammel N. Ross T. L. Lewis S. Olson M. Henciak S. Harris R.. (2021). Comparison of an automated plate assessment system (APAS Independence) and artificial intelligence (AI) to manual plate Reading of methicillin-resistant and methicillin-susceptible *Staphylococcus aureus* CHROMagar surveillance cultures. J. Clin. Microbiol. 59:e0097121. doi: 10.1128/JCM.00971-21, PMID: 34379525 PMC8525556

[ref32] Gao S. Wang H. (2022). Scenario prediction of public health emergencies using infectious disease dynamics model and dynamic Bayes. Futur. Gener. Comput. Syst. 127, 334–346. doi: 10.1016/j.future.2021.09.028, PMID: 34566221 PMC8452458

[ref33] GBD (2019). Antimicrobial resistance collaborators. Global mortality associated with 33 bacterial pathogens in 2019: a systematic analysis for the global burden of disease study 2019. Lancet 400, 2221–2248. doi: 10.1016/S0140-6736(22)02185-7, PMID: 36423648 PMC9763654

[ref35] Goodman K. E. Lessler J. Cosgrove S. E. Harris A. D. Lautenbach E. Han J. H.. (2016). A clinical decision tree to predict whether a Bacteremic patient is infected with an extended-Spectrum β-lactamase-producing organism. Clin. Infect. Dis. 63, 896–903. doi: 10.1093/cid/ciw425, PMID: 27358356 PMC5019284

[ref36] Goodswen S. J. Barratt J. L. N. Kennedy P. J. Kaufer A. Calarco L. Ellis J. T. (2021). Machine learning and applications in microbiology. FEMS Microbiol. Rev. 45:fuab015. doi: 10.1093/femsre/fuab015, PMID: 33724378 PMC8498514

[ref37] Gurvic D. Leach A. G. Zachariae U. (2022). Data-driven derivation of molecular substructures that enhance drug activity in gram-negative Bacteria. J. Med. Chem. 65, 6088–6099. doi: 10.1021/acs.jmedchem.1c01984, PMID: 35427114 PMC9059115

[ref38] Han N. Oh O. H. Oh J. Kim Y. Lee Y. Cha W. C.. (2024). The application of knowledge-based clinical decision support systems to detect antibiotic allergy. Antibiotics (Basel) 13:244. doi: 10.3390/antibiotics13030244, PMID: 38534679 PMC10967561

[ref39] Heinson A. I. Woelk C. H. Newell M.-L. (2015). The promise of reverse vaccinology. Int. Health 7, 85–89. doi: 10.1093/inthealth/ihv002 25733557

[ref40] Hellmich T. R. Clements C. M. El-Sherif N. Pasupathy K. S. Nestler D. M. Boggust A.. (2017). Contact tracing with a real-time location system: a case study of increasing relative effectiveness in an emergency department. Am. J. Infect. Control 45, 1308–1311. doi: 10.1016/j.ajic.2017.08.014, PMID: 28967513 PMC7115342

[ref41] Hirosawa T. Harada Y. Yokose M. Sakamoto T. Kawamura R. Shimizu T. (2023). Diagnostic accuracy of differential-diagnosis lists generated by generative Pretrained transformer 3 Chatbot for clinical vignettes with common chief complaints: a pilot study. Int. J. Environ. Res. Public Health 20:3378. doi: 10.3390/ijerph20043378, PMID: 36834073 PMC9967747

[ref42] Ho C.-S. Jean N. Hogan C. A. Blackmon L. Jeffrey S. S. Holodniy M.. (2019). Rapid identification of pathogenic bacteria using Raman spectroscopy and deep learning. Nat. Commun. 10:4927. doi: 10.1038/s41467-019-12898-9, PMID: 31666527 PMC6960993

[ref43] Horvath L. Hänselmann S. Mannsperger H. Degenhardt S. Last K. Zimmermann S.. (2020). Machine-assisted interpretation of auramine stains substantially increases through-put and sensitivity of microscopic tuberculosis diagnosis. Tuberculosis (Edinb.) 125:101993. doi: 10.1016/j.tube.2020.101993 33010589

[ref44] Howard A. Aston S. Gerada A. Reza N. Bincalar J. Mwandumba H.. (2024). Antimicrobial learning systems: an implementation blueprint for artificial intelligence to tackle antimicrobial resistance. Lancet Digit. Health 6, e79–e86. doi: 10.1016/S2589-7500(23)00221-2, PMID: 38123255

[ref45] Hsu K.-W. Lee W.-B. You H.-L. Lee M. S. Lee G.-B. (2021). An automated and portable antimicrobial susceptibility testing system for urinary tract infections. Lab Chip 21, 755–763. doi: 10.1039/d0lc01315c, PMID: 33503076

[ref46] Hummel P. Braun M. (2020). Just data? Solidarity and justice in data-driven medicine. Life Sci. Soc. Policy 16:8. doi: 10.1186/s40504-020-00101-7, PMID: 32839878 PMC7445015

[ref47] CLSI. Performance Standards for Antimicrobial Susceptibility Testing. 33rd ed.CLSI supplement M100. Clinical and Laboratory Standards Institute; 2023. https://iacld.com/UpFiles/Documents/672a1c7c-d4ad-404e-b10e-97c19e21cdce.pdf [Accessed April 7, 2024]

[ref48] Jiang Y. Luo J. Huang D. Liu Y. Li D. (2022). Machine learning advances in microbiology: a review of methods and applications. Front. Microbiol. 13:925454. doi: 10.3389/fmicb.2022.925454, PMID: 35711777 PMC9196628

[ref49] Keith M. Park de la Torriente A. Chalka A. Vallejo-Trujillo A. McAteer S. P. Paterson G. K.. (2024). Predictive phage therapy for *Escherichia coli* urinary tract infections: cocktail selection for therapy based on machine learning models. Proc. Natl. Acad. Sci. USA 121:e2313574121. doi: 10.1073/pnas.2313574121, PMID: 38478693 PMC10962980

[ref50] Khanna D. Rana P. S. (2019). Ensemble technique for prediction of T-cell *Mycobacterium tuberculosis* epitopes. Interdiscip. Sci. 11, 611–627. doi: 10.1007/s12539-018-0309-0, PMID: 30406342

[ref51] Kleandrova V. V. Speck-Planche A. (2020). The QSAR paradigm in fragment-based drug discovery: from the virtual generation of target inhibitors to multi-scale modeling. Mini Rev. Med. Chem. 20, 1357–1374. doi: 10.2174/1389557520666200204123156, PMID: 32013845

[ref52] Kulshrestha M. Tiwari M. Tiwari V. (2024). Bacteriophage therapy against ESKAPE bacterial pathogens: current status, strategies, challenges, and future scope. Microb. Pathog. 186:106467. doi: 10.1016/j.micpath.2023.106467 38036110

[ref53] Laliwala A. Svechkarev D. Sadykov M. R. Endres J. Bayles K. W. Mohs A. M. (2022). Simpler procedure and improved performance for pathogenic Bacteria analysis with a paper-based Ratiometric fluorescent sensor Array. Anal. Chem. 94, 2615–2624. doi: 10.1021/acs.analchem.1c05021, PMID: 35073053 PMC10091516

[ref54] Lane T. R. Urbina F. Rank L. Gerlach J. Riabova O. Lepioshkin A.. (2022). Machine learning models for *Mycobacterium tuberculosis in vitro* activity: prediction and target visualization. Mol. Pharm. 19, 674–689. doi: 10.1021/acs.molpharmaceut.1c00791, PMID: 34964633 PMC9121329

[ref55] Langford B. J. Branch-Elliman W. Nori P. Marra A. R. Bearman G. (2024). Confronting the disruption of the infectious diseases workforce by artificial intelligence: what this means for us and what we can do about it. Open Forum Infect. Dis. 11:ofae053. doi: 10.1093/ofid/ofae053, PMID: 38434616 PMC10906702

[ref56] Larentzakis A. Lygeros N. (2021). Artificial intelligence (AI) in medicine as a strategic valuable tool. Pan Afr. Med. J. 38:184. doi: 10.11604/pamj.2021.38.184.28197, PMID: 33995790 PMC8106796

[ref57] Larsen P. E. Collart F. R. Dai Y. (2014). Using metabolomic and transportomic modeling and machine learning to identify putative novel therapeutic targets for antibiotic resistant pseudomonad infections. Annu. Int. Conf. IEEE Eng. Med. Biol. Soc. 2014, 314–317. doi: 10.1109/EMBC.2014.6943592, PMID: 25569960

[ref58] Lesosky M. McGeer A. Simor A. Green K. Low D. E. Raboud J. (2011). Effect of patterns of transferring patients among healthcare institutions on rates of nosocomial methicillin-resistant *Staphylococcus aureus* transmission: a Monte Carlo simulation. Infect. Control Hosp. Epidemiol. 32, 136–147. doi: 10.1086/657945 21460468

[ref59] Li R. Shen M. Liu H. Bai L. Zhang L. (2023). Do infrared thermometers hold promise for an effective early warning system for emerging respiratory infectious diseases? JMIR Form Res. 7:e42548. doi: 10.2196/42548, PMID: 37133929 PMC10193206

[ref60] Liu W. Ying N. Mo Q. Li S. Shao M. Sun L.. (2021). Machine learning for identifying resistance features of Klebsiella pneumoniae using whole-genome sequence single nucleotide polymorphisms. J. Med. Microbiol. 70. doi: 10.1099/jmm.0.001474 34812714

[ref61] Lu J. Chen J. Liu C. Zeng Y. Sun Q. Li J.. (2022). Identification of antibiotic resistance and virulence-encoding factors in *Klebsiella pneumoniae* by Raman spectroscopy and deep learning. Microb. Biotechnol. 15, 1270–1280. doi: 10.1111/1751-7915.13960, PMID: 34843635 PMC8966003

[ref62] Mandal S. Tannert A. Löffler B. Neugebauer U. Silva L. B. (2024). Findaureus: an open-source application for locating *Staphylococcus aureus* in fluorescence-labelled infected bone tissue slices. PLoS One 19:e0296854. doi: 10.1371/journal.pone.0296854, PMID: 38295056 PMC10830009

[ref63] Mc Cord-De Iaco K. A. Gesualdo F. Pandolfi E. Croci I. Tozzi A. E. (2023). Machine learning clinical decision support systems for surveillance: a case study on pertussis and RSV in children. Front. Pediatr. 11:1112074. doi: 10.3389/fped.2023.1112074, PMID: 37284288 PMC10239967

[ref64] McGregor J. C. Weekes E. Forrest G. N. Standiford H. C. Perencevich E. N. Furuno J. P.. (2006). Impact of a computerized clinical decision support system on reducing inappropriate antimicrobial use: a randomized controlled trial. J. Am. Med. Inform. Assoc. 13, 378–384. doi: 10.1197/jamia.M2049, PMID: 16622162 PMC1513678

[ref65] Mintz Y. Brodie R. (2019). Introduction to artificial intelligence in medicine. Minim. Invasive Ther. Allied Technol. 28, 73–81. doi: 10.1080/13645706.2019.1575882, PMID: 30810430

[ref66] Nakar A. Pistiki A. Ryabchykov O. Bocklitz T. Rösch P. Popp J. (2022). Detection of multi-resistant clinical strains of *E. coli* with Raman spectroscopy. Anal. Bioanal. Chem. 414, 1481–1492. doi: 10.1007/s00216-021-03800-y, PMID: 34982178 PMC8761712

[ref67] Nguyen M. Brettin T. Long S. W. Musser J. M. Olsen R. J. Olson R.. (2018). Developing an in silico minimum inhibitory concentration panel test for *Klebsiella pneumoniae*. Sci. Rep. 8:421. doi: 10.1038/s41598-017-18972-w, PMID: 29323230 PMC5765115

[ref68] Njage P. M. K. Leekitcharoenphon P. Hald T. (2019). Improving hazard characterization in microbial risk assessment using next generation sequencing data and machine learning: predicting clinical outcomes in shigatoxigenic *Escherichia coli*. Int. J. Food Microbiol. 292, 72–82. doi: 10.1016/j.ijfoodmicro.2018.11.016, PMID: 30579059

[ref69] Oh J. Makar M. Fusco C. McCaffrey R. Rao K. Ryan E. E.. (2018). A generalizable, data-driven approach to predict daily risk of *Clostridium difficile* infection at two large academic health centers. Infect. Control Hosp. Epidemiol. 39, 425–433. doi: 10.1017/ice.2018.16, PMID: 29576042 PMC6421072

[ref70] Ohkusu K. (2000). Cost-effective and rapid presumptive identification of gram-negative bacilli in routine urine, pus, and stool cultures: evaluation of the use of CHROMagar orientation medium in conjunction with simple biochemical tests. J. Clin. Microbiol. 38, 4586–4592. doi: 10.1128/JCM.38.12.4586-4592.2000, PMID: 11101600 PMC87641

[ref71] Paquin P. Durmort C. Paulus C. Vernet T. Marcoux P. R. Morales S. (2022). Spatio-temporal based deep learning for rapid detection and identification of bacterial colonies through lens-free microscopy time-lapses. PLoS Digit. Health 1:e0000122. doi: 10.1371/journal.pdig.0000122, PMID: 36812631 PMC9931332

[ref72] Pérez-Sancho M. Vela A. I. Horcajo P. Ugarte-Ruiz M. Domínguez L. Fernández-Garayzábal J. F.. (2018). Rapid differentiation of *Staphylococcus aureus* subspecies based on MALDI-TOF MS profiles. J. Vet. Diagn. Invest. 30, 813–820. doi: 10.1177/1040638718805537, PMID: 30280650 PMC6505833

[ref73] Periasamy A. (2014). Advanced light microscopy. Methods 66, 121–123. doi: 10.1016/j.ymeth.2014.03.011 24674079

[ref74] Pfeiffer D. U. Stevens K. B. (2015). Spatial and temporal epidemiological analysis in the big data era. Prev. Vet. Med. 122, 213–220. doi: 10.1016/j.prevetmed.2015.05.012, PMID: 26092722 PMC7114113

[ref75] Qiu J. Nie W. Ding H. Dai J. Wei Y. Li D.. (2024). PB-LKS: a python package for predicting phage-bacteria interaction through local K-mer strategy. Brief. Bioinform. 25:bbae010. doi: 10.1093/bib/bbae010, PMID: 38344864 PMC10859729

[ref76] Ramachandran P. S. Ramesh A. Creswell F. V. Wapniarski A. Narendra R. Quinn C. M.. (2022). Integrating central nervous system metagenomics and host response for diagnosis of tuberculosis meningitis and its mimics. Nat. Commun. 13:1675. doi: 10.1038/s41467-022-29353-x, PMID: 35354815 PMC8967864

[ref77] Rapún-Araiz B. Sorzabal-Bellido I. Asensio-López J. Lázaro-Díez M. Ariz M. Sobejano de la Merced C.. (2023). *In vitro* modeling of polyclonal infection dynamics within the human airways by *Haemophilus influenzae* differential fluorescent labeling. Microbiol. Spectr. 11:e0099323. doi: 10.1128/spectrum.00993-23, PMID: 37795992 PMC10714817

[ref78] Rawal K. Sinha R. Abbasi B. A. Chaudhary A. Nath S. K. Kumari P.. (2021). Identification of vaccine targets in pathogens and design of a vaccine using computational approaches. Sci. Rep. 11:17626. doi: 10.1038/s41598-021-96863-x, PMID: 34475453 PMC8413327

[ref79] Rees C. Müller B. (2022). All that glitters is not gold: trustworthy and ethical AI principles. AI Ethics 3, 1241–1254. doi: 10.1007/s43681-022-00232-x, PMID: 36406882 PMC9667859

[ref80] Rhodes N. J. Rohani R. Yarnold P. R. Pawlowski A. E. Malczynski M. Qi C.. (2023). Machine learning to stratify methicillin-resistant *Staphylococcus aureus* risk among hospitalized patients with community-acquired pneumonia. Antimicrob. Agents Chemother. 67:e0102322. doi: 10.1128/aac.01023-22, PMID: 36472425 PMC9872682

[ref81] Rodrigues Lopes I. Alcantara L. M. Silva R. J. Josse J. Vega E. P. Cabrerizo A. M.. (2022). Microscopy-based phenotypic profiling of infection by *Staphylococcus aureus* clinical isolates reveals intracellular lifestyle as a prevalent feature. Nat. Commun. 13:7174. doi: 10.1038/s41467-022-34790-9, PMID: 36418309 PMC9684519

[ref82] Santa Maria J. P. Park Y. Yang L. Murgolo N. Altman M. D. Zuck P.. (2017). Linking high-throughput screens to identify MoAs and novel inhibitors of *Mycobacterium tuberculosis* Dihydrofolate reductase. ACS Chem. Biol. 12, 2448–2456. doi: 10.1021/acschembio.7b00468, PMID: 28806050 PMC6298432

[ref83] Schwartz I. S. Link K. E. Daneshjou R. Cortés-Penfield N. (2024). Black box warning: large language models and the future of infectious diseases consultation. Clin. Infect. Dis. 78, 860–866. doi: 10.1093/cid/ciad633, PMID: 37971399 PMC11006107

[ref84] Senescau A. Kempowsky T. Bernard E. Messier S. Besse P. Fabre R.. (2018). Innovative DendrisChips® Technology for a Syndromic Approach of *in vitro* diagnosis: application to the respiratory infectious diseases. Diagnostics (Basel) 8:77. doi: 10.3390/diagnostics8040077, PMID: 30423863 PMC6316573

[ref85] Shen Y. Yuan K. Chen D. Colloc J. Yang M. Li Y.. (2018). An ontology-driven clinical decision support system (IDDAP) for infectious disease diagnosis and antibiotic prescription. Artif. Intell. Med. 86, 20–32. doi: 10.1016/j.artmed.2018.01.003, PMID: 29433958

[ref86] Sherry N. L. Horan K. A. Ballard S. A. Gonҫalves da Silva A. Gorrie C. L. Schultz M. B.. (2023). An ISO-certified genomics workflow for identification and surveillance of antimicrobial resistance. Nat. Commun. 14:60. doi: 10.1038/s41467-022-35713-4, PMID: 36599823 PMC9813266

[ref87] Stracy M. Snitser O. Yelin I. Amer Y. Parizade M. Katz R.. (2022). Minimizing treatment-induced emergence of antibiotic resistance in bacterial infections. Science (New York, N.Y.) 375, 889–894. doi: 10.1126/science.abg9868, PMID: 35201862 PMC7612469

[ref88] Tagliaferri T. L. Jansen M. Horz H.-P. (2019). Fighting pathogenic Bacteria on two fronts: phages and antibiotics as combined strategy. Front. Cell. Infect. Microbiol. 9:22. doi: 10.3389/fcimb.2019.00022, PMID: 30834237 PMC6387922

[ref89] Tilton C. S. Johnson S. W. (2019). Development of a risk prediction model for hospital-onset *Clostridium difficile* infection in patients receiving systemic antibiotics. Am. J. Infect. Control 47, 280–284. doi: 10.1016/j.ajic.2018.08.021 30318399

[ref90] Ting Sim J. Z. Fong Q. W. Huang W. Tan C. H. (2023). Machine learning in medicine: what clinicians should know. Singapore Med. J. 64, 91–97. doi: 10.11622/smedj.2021054, PMID: 34005847 PMC10071847

[ref91] Váradi L. Luo J. L. Hibbs D. E. Perry J. D. Anderson R. J. Orenga S.. (2017). Methods for the detection and identification of pathogenic bacteria: past, present, and future. Chem. Soc. Rev. 46, 4818–4832. doi: 10.1039/c6cs00693k, PMID: 28644499

[ref92] Viertel T. M. Ritter K. Horz H.-P. (2014). Viruses versus bacteria-novel approaches to phage therapy as a tool against multidrug-resistant pathogens. J. Antimicrob. Chemother. 69, 2326–2336. doi: 10.1093/jac/dku173, PMID: 24872344

[ref93] Villarroel J. Kleinheinz K. A. Jurtz V. I. Zschach H. Lund O. Nielsen M.. (2016). HostPhinder: a phage host prediction tool. Viruses 8:116. doi: 10.3390/v8050116, PMID: 27153081 PMC4885074

[ref94] Volynets G. P. Usenko M. O. Gudzera O. I. Starosyla S. A. Balanda A. O. Syniugin A. R.. (2022). Identification of dual-targeted *Mycobacterium tuberculosis* aminoacyl-tRNA synthetase inhibitors using machine learning. *Future*. Med. Chem. 14, 1223–1237. doi: 10.4155/fmc-2022-0085, PMID: 35876255

[ref95] Waddington C. Carey M. E. Boinett C. J. Higginson E. Veeraraghavan B. Baker S. (2022). Exploiting genomics to mitigate the public health impact of antimicrobial resistance. Genome Med. 14:15. doi: 10.1186/s13073-022-01020-2, PMID: 35172877 PMC8849018

[ref96] Walsh T. R. Gales A. C. Laxminarayan R. Dodd P. C. (2023). Antimicrobial resistance: addressing a global threat to humanity. PLoS Med. 20:e1004264. doi: 10.1371/journal.pmed.1004264, PMID: 37399216 PMC10317217

[ref97] Wang H. Ceylan Koydemir H. Qiu Y. Bai B. Zhang Y. Jin Y.. (2020). Early detection and classification of live bacteria using time-lapse coherent imaging and deep learning. Light Sci. Appl. 9:118. doi: 10.1038/s41377-020-00358-9, PMID: 32685139 PMC7351775

[ref98] Wang H.-Y. Chung C.-R. Wang Z. Li S. Chu B.-Y. Horng J.-T.. (2021). A large-scale investigation and identification of methicillin-resistant *Staphylococcus aureus* based on peaks binning of matrix-assisted laser desorption ionization-time of flight MS spectra. Brief. Bioinform. 22:bbaa138. doi: 10.1093/bib/bbaa138, PMID: 32672791 PMC8138823

[ref99] Wang M. Wei Z. Jia M. Chen L. Ji H. (2022). Deep learning model for multi-classification of infectious diseases from unstructured electronic medical records. BMC Med. Inform. Decis. Mak. 22:41. doi: 10.1186/s12911-022-01776-y, PMID: 35168624 PMC8848865

[ref100] Wells J. Grant R. Chang J. Kayyali R. (2021). Evaluating the usability and acceptability of a geographical information system (GIS) prototype to visualise socio-economic and public health data. BMC Public Health 21:2151. doi: 10.1186/s12889-021-12072-1, PMID: 34819037 PMC8611402

[ref101] Wieser A. Schneider L. Jung J. Schubert S. (2012). MALDI-TOF MS in microbiological diagnostics-identification of microorganisms and beyond (mini review). Appl. Microbiol. Biotechnol. 93, 965–974. doi: 10.1007/s00253-011-3783-4, PMID: 22198716

[ref102] Wilson M. L. (2015). Diagnostic microbiology: the accelerating transition from culture-based to molecular-based methods. Am. J. Clin. Pathol. 143, 766–767. doi: 10.1309/AJCPIC9GPLHCV1NT 25972317

[ref103] Wong F. De La Fuente-Nunez C. Collins J. J. (2023). Leveraging artificial intelligence in the fight against infectious diseases. Science 381, 164–170. doi: 10.1126/science.adh1114, PMID: 37440620 PMC10663167

[ref104] Yan Y. Chen C. Liu Y. Zhang Z. Xu L. Pu K. (2021). Application of machine learning for the prediction of etiological types of classic fever of unknown origin. Front. Public Health 9:800549. doi: 10.3389/fpubh.2021.800549, PMID: 35004599 PMC8739804

[ref105] Yi Q. Cai D. Xiao M. Nie M. Cui Q. Cheng J.. (2019). Direct antimicrobial susceptibility testing of bloodstream infection on SlipChip. Biosens. Bioelectron. 135, 200–207. doi: 10.1016/j.bios.2019.04.003, PMID: 31026774

[ref106] Ying C. Li X. Lv S. Du P. Chen Y. Fu H.. (2022). T-SPOT with CT image analysis based on deep learning for early differential diagnosis of nontuberculous mycobacteria pulmonary disease and pulmonary tuberculosis. Int. J. Infect. Dis. 125, 42–50. doi: 10.1016/j.ijid.2022.09.031, PMID: 36180035

[ref107] Youn J. Rai N. Tagkopoulos I. (2022). Knowledge integration and decision support for accelerated discovery of antibiotic resistance genes. Nat. Commun. 13:2360. doi: 10.1038/s41467-022-29993-z, PMID: 35487919 PMC9055065

[ref108] Younes S. Al-Sulaiti A. Nasser E. A. A. Najjar H. Kamareddine L. (2020). Drosophila as a model organism in host-pathogen interaction studies. Front. Cell. Infect. Microbiol. 10:214. doi: 10.3389/fcimb.2020.00214, PMID: 32656090 PMC7324642

[ref109] Yu J. Lin Y.-T. Chen W.-C. Tseng K.-H. Lin H.-H. Tien N.. (2023b). Direct prediction of carbapenem-resistant, carbapenemase-producing, and colistin-resistant *Klebsiella pneumoniae* isolates from routine MALDI-TOF mass spectra using machine learning and outcome evaluation. Int. J. Antimicrob. Agents 61:106799. doi: 10.1016/j.ijantimicag.2023.106799, PMID: 37004755

[ref110] Yu J. Lin H.-H. Tseng K.-H. Lin Y.-T. Chen W.-C. Tien N.. (2023a). Prediction of methicillin-resistant Staphylococcus aureus and carbapenem-resistant *Klebsiella pneumoniae* from flagged blood cultures by combining rapid Sepsityper MALDI-TOF mass spectrometry with machine learning. Int. J. Antimicrob. Agents 62:106994. doi: 10.1016/j.ijantimicag.2023.106994, PMID: 37802231

[ref111] Zhang Y.-M. Tsao M.-F. Chang C.-Y. Lin K.-T. Keller J. J. Lin H.-C. (2023). Rapid identification of carbapenem-resistant *Klebsiella pneumoniae* based on matrix-assisted laser desorption ionization time-of-flight mass spectrometry and an artificial neural network model. J. Biomed. Sci. 30:25. doi: 10.1186/s12929-023-00918-2, PMID: 37069555 PMC10108464

